# Preliminary Results of a Multicenter Randomized Clinical Trial for Laparoscopic Repair of Pelvic Organ Prolapse: Sacropexy vs. Laparoscopic Lateral Suspension

**DOI:** 10.3390/jcm14062069

**Published:** 2025-03-18

**Authors:** Isabel Ñíguez-Sevilla, María Luisa Sánchez-Ferrer, Vicente Luis Ruiz-Cotorruelo, Maciej Wilczak, Karolina Chmaj-Wierzchowska, Juan Antonio Solano-Calvo, María Elena Pérez-Muñuzuri, Juan Raúl Salinas-Peña, Julián Jesús Arense-Gonzalo

**Affiliations:** 1 Department of Obstetrics & Gynaecology, “Virgen de la Arrixaca” University Clinical Hospital, 30120 Murcia, Spain; isnise@gmail.com (I.Ñ.-S.); ruiz_vic@icloud.com (V.L.R.-C.); 2 Biomedical Research Institute of Murcia (IMIB-Arrixaca), University Clinical Hospital “Virgen de la Arrixaca”, 30120 Murcia, Spain; julianjesus.arense@um.es; 3 Department of Maternal and Child Health and Minimally Invasive Surgery, Poznan University of Medical Science, Polna 33, 60-535 Poznań, Poland; mwil@gpsk.ump.edu.pl (M.W.); karolinachmaj@poczta.onet.pl (K.C.-W.); 4 University Hospital Príncipe de Asturias, Alcalá de Henares, 28805 Madrid, Spain; jsolanoc@sego.es; 5 University Clinical Hospital Santiago de Compostela, 15701 Santiago, Spain; elena_munuzuri@hotmail.com; 6 San Juan de Reus University Hospital, Avinguda del Doctor Josep Laporte, 2, 43204 Reus, Spain; jrsalinas2@gmail.com; 7 Division of Preventive Medicine and Public Health, Department of Public Health Sciences, University of Murcia School of Medicine, El Palmar, 30120 Murcia, Spain

**Keywords:** laparoscopic, lateral suspension, sacropexy, pelvic organ prolapse

## Abstract

**Background:** Laparoscopic sacropexy (SCL) is the gold standard technique for the correction of apical pelvic organ prolapse (POP). However, other easier laparoscopic techniques, such as laparoscopic lateral suspension (LLS), have become popular. **Methods:** We conducted a multicenter randomized study of patients undergoing laparoscopic repair of apical and anterior prolapse. Patients were randomized into two groups: LLS vs. SCL. A non-inferiority study was proposed, in which the null hypothesis was that the difference in the proportion of therapeutic failures among women who undergo LLS compared to SCL is ≥15%. It was necessary to include 182 participants to detect a risk difference of 15% after one year with a statistical power of 0.80. **Results:** We recruited 176 women, of whom 106 patients underwent surgery with a follow-up between 1 and 12 months. There were no differences in basal characteristics. Regarding physical examination, there were no differences at stages III-IV in the POP-Q or the symptom scales in both groups. Concerning the post-surgical results, there were no failures detected in the physical examination in any group. There were no differences in the points of the POP-Q, the symptom scales, or the body image scale. We only found significant differences in the operative time, which was shorter for the LLS. **Conclusions:** Although these are preliminary results, since the sample includes 106 patients and the follow-up time is a limited period at the moment, we did not find any post-surgical differences between the two techniques. However, it will be necessary to complete the trial to draw relevant conclusions.

## 1. Introduction

Pelvic organ prolapse (POP) is a common benign condition or pathology. More than 24% of adult women have symptoms of pelvic floor disorders [1]. The percentage of parous women with POP is 40% to 60% [2]. Most women with prolapse experience symptoms that negatively impact their daily activities, sexual function, and quality of life. The risk of undergoing surgery for genital prolapse throughout a woman’s lifespan is 11–19% [3,4,5], and almost a third of those who undergo surgery will require further surgery for recurrence of the prolapse [6]. Surgical treatment of genital prolapse consumes an important portion of health resources worldwide, and it was the most frequently performed surgical procedure in women over 70 years of age between 1979 and 2006 [7].

A wide variety of surgical techniques for the treatment of POP have been described; however, the level of evidence regarding the surgical treatment of prolapse is very limited, with few prospective, comparative, and randomized studies [2,8]. In addition, there is little correlation between anatomical and functional results, and although functional results have the greatest impact on patients’ quality of life, they are the most poorly evaluated area.

Abdominal sacropexy (SCL) (the laparoscopic approach is preferred) is considered the gold standard technique for apical prolapse, with few recurrences compared to vaginal procedures [2,9]. It is based on the suspension of the uterus, cervix, or the vaginal dome from the anterior vertebral ligament of the sacrum (promontory) by the interposition of a prosthetic material (synthetic mesh), which reinforces the anterior and posterior (fibromuscular) walls of the vagina. Laparoscopic sacropexy is a highly complex technique that requires advanced laparoscopic skills [10]. An extensive and deep dissection of the posterior compartment involves an increased risk of rectal perforation and the possibility of injury to the rectal irrigation and innervation with potential functional effects on defecation. The necessary learning curve and the fear of exposing patients to excessive morbidity during this learning curve has resulted in a limited and very gradual inclusion of laparoscopic sacropexy among the usual techniques for the treatment of apical prolapse. Several studies have analyzed the number of interventions necessary to technically master the surgical procedure and reduce operative time, but in general, it is between 18 and 40 interventions [11,12,13].

In addition, given the complexity of the sacropexy technique, other laparoscopic prolapse correction alternatives have emerged, such as pectopexy [14] and the laparoscopic lateral suspension (LLS) technique described by Dubuisson [15]. With the LLS technique, the mesh is fixed to the uterus or the vaginal vault, and the arms of the mesh are passed under the peritoneum (in an extraperitoneal manner) without being fixed to any anatomical structure. LLS is a standardized, simpler, and faster technique than sacropexy and requires fewer sutures, which allows for a shorter learning curve. Nowadays, there is extensive literature on this technique [15,16,17,18,19,20,21], but randomized comparative clinical trials are necessary to compare the post-surgical results between sacropexy and LLS.

For that reason, we designed a multicenter randomized study to determine if the laparoscopic lateral suspension (LLS) technique can offer anatomical and functional results that are not inferior to those of the conventional surgical technique (sacropexy), minimize possible intraoperative complications, and reduce the long and specific learning curve of sacropexy.

## 2. Materials and Methods

A multicenter randomized study of patients undergoing laparoscopic repair of severe apical and anterior prolapse was carried out. We divided the patients into two groups:

- Group A: lateral laparoscopic suspension (LLS).
- Group B: Sacropexy (SCL) without posterior mesh fixation on the puborectalis muscle.

The surgical techniques are described in Supplementary Materials.

In both groups, it is possible to perform hysteropexy, supracervical hysterectomy, or repair of vaginal vault prolapse after hysterectomy (it depends on each patient’s needs).

The exclusion criteria for hysteropexy in both groups was the contraindications for uterine preservation: uterine pathology, including fibroids, adenomyosis, and endometrial pathology; cervical lesions; post-menopausal bleeding; cervical elongation (defined as POP-Q [22] Point C minus Point D ≥ 4); ovarian/tube cancer risk (BRCA 1&2), endometrial cancer risk, Lynch syndrome, Tamoxifen treatment, and inability to follow a gynecological cancer prevention program.

This study was conducted in accordance with the Declaration of Helsinki and approved by the Ethics Committee of CEIm Hospital Virgen de la Arrixaca (protocol code 2022-3-8-HCUVA and date of approval: 26 April 2022). Written informed consent was obtained from all subjects involved in this study. This trial was registered in ClinicalTrials.gov, with the Identifier NCT06815731. The full protocol is described in Annex 2.

Randomization system: Random Allocation Rule, a free-source software package version 3.0.2 in randomizeR: A Package for the Assessment and Implementation of Randomization in Clinical Trials (Uschner et al.), was used. The allocator accessed the software using a personal password and had a list of the admitted patients and their randomization.

A non-inferiority study was proposed, in which the null hypothesis (H0) was that the difference in the proportion of treatment failures among women who undergo laparoscopic bilateral suspension (LLS) (Group A) compared to the proportion of anatomical and/or functional failures among women who undergo sacropexy without the fixation of the posterior mesh on puborectalis muscle (Group B) is 15% or more (non-inferiority margin). A non-inferiority margin of 15% was used because minor differences are not considered clinically relevant. It was necessary to include 182 participants (91 per group) to detect a risk difference of 15% (8% failure for the LLS group versus 23% failure for the sacropexy group) after 1 year of follow-up with a statistical power of 0.80.

The inclusion criteria included patients with stages II-IV primary or recurrent prolapse affecting the anterior or middle vaginal compartment with or without minimal posterior defect (stage I) according to the POP-Q.

The exclusion criteria included a history of abdominal prolapse reconstructive surgery, a history of prolapse reconstructive surgery with vaginal meshes, stage I according to the POP-Q classification or asymptomatic prolapse, medical contraindication for general anesthesia, patient preference for the vaginal surgical approach, or the patient did not wish to participate in this study.

The primary outcome was treatment failure, a composite measure that includes any of the following: (A) new treatment for prolapse (pessary placement or surgery) and (B) anatomical results, defined as any POP-Q [22] measurement beyond the hymen. For the primary analysis, this outcome was assessed cumulatively so that once a participant met any of the failure criteria, her outcome was classified as treatment failure.

The secondary objectives were to assess whether there are differences in complications, adverse events, and individual anatomical measures on the POP-Q exam. We also assessed the presence, severity, and impact of symptoms of or discomfort from prolapse and urinary, intestinal, and pain symptoms, as measured by the PFDI-20 [23] and PISQ-12 [24] questionnaires and the body image scale [25], between the lateral laparoscopic suspension (LLS) (Group A) and sacropexy (Group B).

A pre-surgery visit and 3 follow-up visits (1 month, 6 months, and 1 year post-surgery) were planned. The trial began in October 2023, and it is ongoing, but the authors wanted to perform a preliminary analysis to determine if it is ethical to continue with this study until the number of participants in both techniques is complete.

The collection of follow-up variables was conducted by a specialist who did not know which surgical technique was performed for each patient to eliminate the possibility of bias in the assessment of the post-surgical results.

## 3. Results

**Figure 1:**
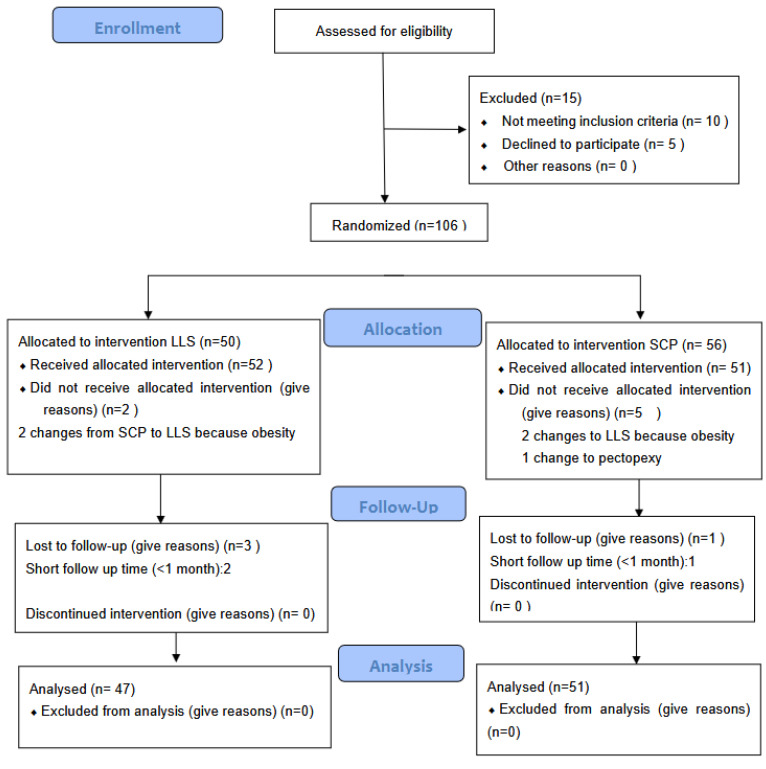
Flowchart.

At the moment, we have included 176 patients, and 106 patients have been operated on as follows: 50 LLS and 56 SCL, with a follow-up between 1 and 12 months (Figure 1).

There were no differences in baseline demographic characteristics of the patients between the two groups (Table 1). We only found differences in the mean age (but the difference is not clinically relevant: 57.9 years for LLS vs. 54.7 years for SCL). No statistically significant differences were found in BMI (body mass index), multiparity, vaginal and instrumented deliveries, macrosomic fetuses, previous constipation, chronic sports or exertion, previous abdominal or vaginal hysterectomy, or previous vaginal surgeries. Regarding the physical examination, all patients included in both groups had no differences in stages III-IV in the POP-Q classification. Concerning the symptom scales, we also found no significant differences between the groups in the mean values of POPDI-6, CRAD-8, UDI-6, and PISQ-12.

Regarding the post-surgical results, we found statistically significant differences in the total surgical time, which was lower for LLS (76.1 ± 58.6 vs. 164.7 ± 84.9 min, *p* < 0.001); however, there were no differences in the time spent performing the hysterectomy in both groups. Regarding pain on the first postoperative day, assessed using the visual analog scale, there were no significant differences. None of the patients in either group had a major postoperative complication. There were no failures in the physical examination in any group. There were no differences in the points of the POP-Q. There were also no significant differences after surgery in the symptom scales (neither in the POPDI-6, CRAD-8, UDI 6, nor PISQ-12) or the body image scale (Table 2). 

## 4. Discussion

Laparoscopic sacropexy (SCL) is considered the gold standard technique for apical prolapse, but new alternative surgical techniques have become popular in recent years. However, the new techniques need to be compared with the gold standard technique. In addition, there is little correlation between anatomical and functional results, and although functional results have the greatest impact on patients’ quality of life, they are the most poorly evaluated areas.

To date, there are very few published studies that have compared SCL vs. LLS. A group at Pisa University [20] conducted a prospective, open-label, multicenter, non-inferiority trial with 300 patients who underwent SCL (n = 200) or LLS (n = 100) for the treatment of apical prolapse. At the 12-month follow-up, no differences in the objective cure rate of the apical prolapse were found. Another weakness was the design, including they performed hysterectomies in all the SCL patients and hysteropexies in all the patients in the LLS group. Furthermore, robotic and laparoscopic surgeries were analyzed without stratifying the results. The most important difference with our study is that the study was not a randomized trial.

In 2024, a randomized controlled clinical trial was published by a Turkish group [26] with a modest sample size (22 patients in each group). Hysteropexy with LLS and sacrohysteropexy were compared. Previous hysterectomy was an exclusion criterion. One of their weaknesses was the use of V-shaped tailored mesh (2 × 25 cm^2^) and not the pre-shaped mesh marketed for the standardized technique. Another weakness was that the patient selection criteria included women with stage II or higher apical prolapse with or without anterior prolapse; however, the standard technique includes dissection of the vesicovaginal space to correct the anterior prolapse, as in SCL.

The only randomized trial conducted to date with a larger sample size included 89 patients (46 LLS and 43 SCL), but the authors performed hysterectomies at the same time for all the patients [27]. In this study, the anatomic results were similar for both techniques, with no differences in the operative time.

The LLS technique represents a significant advance in the field of reconstructive surgery of POP, and it is a good alternative to SCL. As with any surgical technique, continuous research is needed to improve the procedure, optimize its results, and better define its indications. LLS is a valuable new tool, with increasing evidence in the literature suggesting that it can be used to treat advanced POP. It expands the surgeon’s surgical arsenal and may provide the opportunity to better tailor the type of suspension depending on the type of prolapse. In fact, based on the experience of the participants in the Delphi process [19], LLS is more effective in correcting advanced anterior prolapse compared to SCL. Therefore, if this is confirmed by future trials, LLS could be the alternative to SCL in predominantly apical and anterior defects, while SCL may be more appropriate for the management of prolapses where anterior, apical, and posterior defects predominate.

Mastery of LLS allows the management of the rare cases in which the sacral promontory is difficult to access or has vascular anatomical variations that complicate the dissection. As an additional advantage, the surgical skills required to perform LLS are simpler than those required to perform SCL, as confirmed by the expert panel [19].

The main strength of our study is its design; it is a multicenter and randomized study with a large sample size. In addition, our study presents a comparative analysis of two different abdominal apical POP repairs: LLS and SCL. Additionally, we collected quality-of-life questionnaires to assess subjective outcomes and performed a systematic assessment of the lower urinary tract, the lower gastrointestinal tract, pelvic organ prolapse symptoms, and sexuality and body image scales. The main limitation of this study was that it presented preliminary results; therefore, we need to finish this study to draw relevant conclusions. Another one of the weaknesses of this study could be that it was designed with the initial follow-up to be completed 1 year after surgery. However, perhaps at the end of this study, we will consider extending the follow-up period to 2 years to be able to detect late treatment failures and not just early ones.

## 5. Conclusions

Although the presented results are preliminary because the sample includes 106 patients and the follow-up time is short, at the moment, we have not found post-surgical differences in treatment failure, symptoms, or physical examinations after the operation between the two techniques. We only found significant differences with a shorter surgical time for LLS. In short, it is necessary to extend this study to other centers and complete the clinical trial as soon as possible to be able to draw relevant conclusions.

## Figures and Tables

**Table 1:** Preoperative demographic characteristics.

Technique	LLS			Sacropexy			*p*-Value
	n	Mean	Median (p25–p75)	n	Mean	Median (p25–p75)	
Age	50	57.9 ± 9.5	59.0 (50.0–67.0)	56	54.7 ± 10.3	53.0 (47.0–61.8)	***0.047***
BMI	49	26.2 ± 4.8	25.2 (22.7–28.3)	56	27.0 ± 3.9	26.5 (24.5–29.8)	0.122
n° Pregnancy	50	2.6 ± 1.0	2.0 (2.0–3.0)	56	2.7 ± 1.4	2.0 (2.0–3.0)	0.886
n° Vaginal delivery	50	2.2 ± 1.0	2.0 (2.0–3.0)	56	2.3 ± 1.4	2.0 (1.3–3.0)	0.567
Stage of POP Q ≥ II n (%)							
II		1 (2.6%)			2.0 (4.7%)		0.615
III-IV		38.0 (97.4%)			31.0 (95.3%)		
Questionnaire PFDI-20							
POPDI-6 (0–24)	40	12.4 ± 5.8	12.5 (7.3–16.0)	37	14.5 ± 6.2)	14.0 (11.0–19.5)	0.137
CRADI-8 (0–32)	41	12.0 ± 24.8	8.0 (4.5–10.5)	36	8.1 ± 4.7)	8.0 (4.3–11.8)	0.616
UDI-6 (0–24)	41	13.1 ± 6.3	13.0 (9.0–18.0)	36	12.7 ± 6.0)	13.0 (9.0–17.8)	0.761
PISQ-12 (0–48)	38	28.7 ± 9.0	29.0 (22.8–36.0)	33	29.2 ± 9.3)	28.0 (23.5–36.5)	0.917
Body image scale n (%)							
normal		9 (30.0%)			4.0 (28.6%)		
abnormal		21.0 (70%)			10.0 (71.4%)		0.923
POP-Q points							
Aa	48	1.6 ± 1.0	2.0 (1.0–2.0)	51	2.0 ± 1.2	2.0 (1.5–3.0)	***0.020***
Ba	48	1.6 ± 1.2	1.5 (1.0–2.0)	51	2.5 ± 2.3	3.0 (1.0–4.0)	***0.006***
C o D	48	1.5 ± 1.6	2.0 (0.0–2.4)	51	2.8 ± 2.4	2.0 (1.0–4.0)	***0.017***
Ap	47	−1.1 ± 1.4	−1.0 (−2.0–0.0)	50	−0.7 ± 2.0	−1.0 (−2.0–1.0)	0.528
Bp	44	−1.3 ± 1.7	−2.0 (−3.0–0.0)	50	−0.5 ± 3.0	−1.0 (−2.1–0.0)	0.172
gh	47	4.7 ± 1.0	4.5 (4.0–5.0)	50	5.0 ± 1.3	5.0 (4.0–6.0)	0.060
pb	45	2.8 ± 1.2	3.0 (2.0–3.5)	50	3.0 ± 0.9	3.0 (2.0–3.5)	0.469
tvl	47	7.4 ± 1.0	7.0 (7.0–8.0)	49	7.8 ± 1.3	8.0 (7.0–9.0)	0.131

BMI: body mass index; LLS: laparoscopic lateral suspension; SCL: sacropexy; POP-Q: pelvic organ prolapse quantification.

**Table 2:** Post-surgical results.

Technique	LLS			Sacropexy				
	N	Mean	Median (p25–p75)	N	Mean	Median (p25–p75)	*p*-Value	95% CI Mean Differences
Operative time (min)	47	76.1 ± 58.6	85.0 (0.1–110.0)	51	164.7 + 84.9	180.0 (140.0–210.0)	***<0.001***	***−88.6 [−118.1, −59.1]***
Other surgery times (min)	23	8.0 ± 11.7	0.0 (0.0–15.0)	10	5.5 + 11.2	0.0 (0.0–10.0)	0.466	2.5 [−6.4, 11.5]
Surgery complications	45	1.1 ± 7.2	0.0 (0.0–0.0)	49	1.1 + 7.9	0.0 (0.0–0.0)	0.964	−0.1 [−3.1, 3.0]
Hemoglobin 24 h after surgery	44	11.7 ± 3.9	11.9 (8.6–12.5)	49	11.5 + 2.5	11.5 (10.1–12.6)	0.969	−4526.8 [−9282.3, 228.7]
VAS: pain 1° day after surgery (0–10)	22	3.5 ± 2.1	4.0 (1.8–5.0)	20	3.6 ± 2.2	3.0 (2.0–5.0)	0.929	−0.1 [−1.5, 1.2]
Questionnaire PFDI-20:								
POPDI-6 (0–24)	33	5.5 ± 4.9	6.0 (1.0–8.0)	41	5.1 ± 5.5	4.0 (0.5–8.0)	0.594	0.4 [−2.1, 2.8]
CRADI-8 (0–32)	33	4.7 ± 4.9	3.0 (1.5–6.0)	41	6.4 ± 5.8	4.0 (2.0–10.5)	0.274	−1.7 [−4.2, 0.8]
UDI-6 (0–24)	33	5.9 ± 4.5	6.0 (1.5–10.0)	41	7.5 ± 6.1	6.0 (2.5–11.0)	0.407	−1.6 [−4.2, 0.9]
PISQ-12 (0–48)	20	20.7 ± 11.7	17.0 (13.0–30.8)	31	27.0 ± 11.0	27.0 (18.0–38.0)	0.056	−6.4 [−12.9, 0.2]
Body image scale n (%)								
normal		12 (93.3%)			9 (81.8%)		0.576	10.49% [−16.52%, 37.50%]
abnormal		1 (7.7%)			2 (18.2%)			
POP-Q points								
Aa	35	−2.2 + 1.3	−2.0(−3.0–1.5)	42	−2.3 + 1.1	−3.0(−3.0–2.0)	0.377	−0.1 [−0.4, 0.2]
Ba	35	−2.8 ± 1.4	−3.0 (−3.0–2.0)	42	−2.3 ± 1.1	−3.0 (−3.0–2.0)	0.077	0.2 [−0.4, 0.7]
C o D	35	−4.0 ± 3.5	−5.0 (−6.0–1.0)	42	−5.0 ± 2.6	−6.0 (−7.0–4.4)	0.161	−0.6 [−1.1, 0.0]
Ap	34	−1.8 ± 1.3	−2.0 (−3.0–1.0)	41	−2.1 ± 1.2	−3.0 (−3.0–1.5)	0.191	1.0 [−0.4, 2.4]
Bp	34	−2.7 ± 1.2	−3.0 (−3.0–2.0)	41	−2.1 ± 1.3	−3.0 (−3.0–1.3)	0.189	0.3 [−0.3, 0.9]
gh	35	4.0 ± 1.0	4.0 (3.0–5.0)	41	4.2 ± 1.2	4.0 (3.5–5.0)	0.452	−0.5 [−1.1, 0.0]
pb	34	3.0 ± 0.8	3.0 (2.0–3.5)	41	2.8 ± 1.1	3.0 (2.0–3.3)	0.539	0.2 [−0.3, 0.7]
tvl	35	7.6 ± 1.0	8.0 (7.0–8.0)	41	8.1 ± 1.2	8.0 (7.0–9.0)	0.066	−0.5 [−1.0, 0.0]

LLS: laparoscopic lateral suspension; SCL: sacropexy; POP-Q: pelvic organ prolapse quantification.

## Data Availability

All the data used to support the findings of this study are included in this article.

## Supplementary Materials

The following supporting information can be downloaded at: https://www.mdpi.com/article/10.3390/jcm14062069/s1, Surgical technique: Groups A and B.

## Abbreviations

The following abbreviations are used in this manuscript:

POP	pelvic organ prolapse
SCL	sacropexy (laparoscopic)
LLS	laparoscopic lateral suspension
POPQ	pelvic organ prolapse quantification
PFDI-20	Pelvic Floor Distress Inventory Questionnaire-Short Form 20
POPDI-6	Pelvic Organ Prolapse Distress Inventory
CRAD-8	Colorectal–Anal Distress Inventory
UDI-6	Urinary Distress Inventory
PISQ-12	Pelvic Organ Prolapse/Urinary Incontinence Sexual Function Questionnaire
BMI	body mass index
