# Supplementary material for: Preliminary Results of a Multicenter Randomized Clinical Trial for Laparoscopic Repair of Pelvic Organ Prolapse: Sacropexy vs. Laparoscopic Lateral Suspension
Source: J Clin Med. 2025 Mar 18;14(6):2069. doi: 10.3390/jcm14062069 (PMC11943180; doi:10.3390/jcm14062069)
Supplement: Supplementary file 1 [file jcm-14-02069-s001.zip › jcm-3491574-supplementary.pdf]

Surgical technique:

Group A: Hysteropexy or colpopexy with Dubuisson laparoscopic bilateral suspension

1. Placement of 4 lateral trocars from the cranial finger to the anterior superior iliac spine.

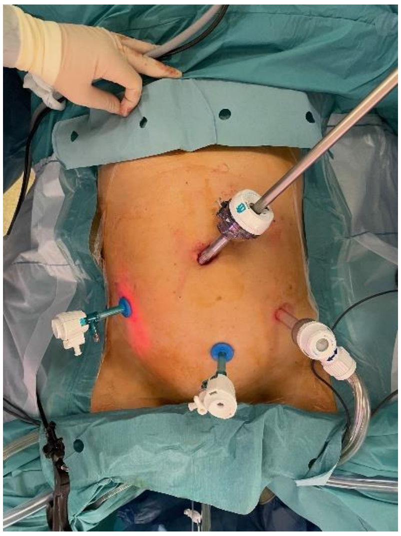

2. Preparation of the vesicovaginal space. It may help to place the valve in the anterior vagina.

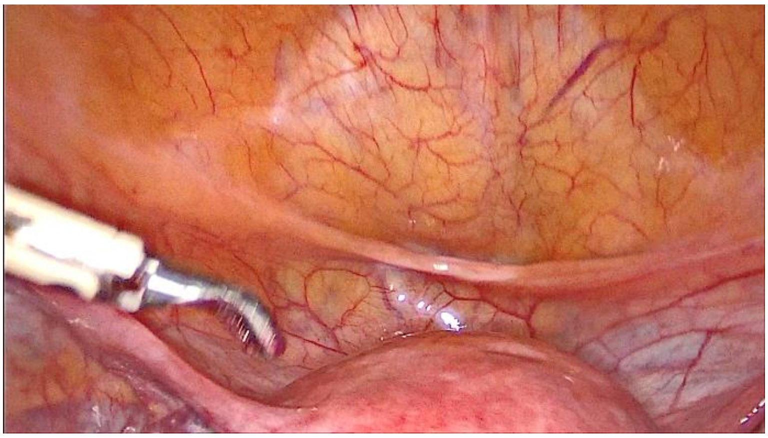

3. Opening of the peritoneum between the bladder and uterus.

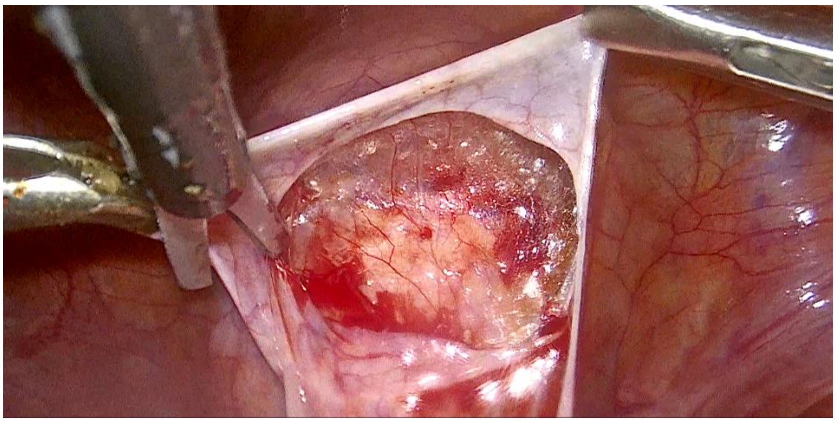

4. Preparation of the vesicovaginal space. Progress to the trigone (6 cm), but not beyond that distally to prevent voiding dysfunctions.

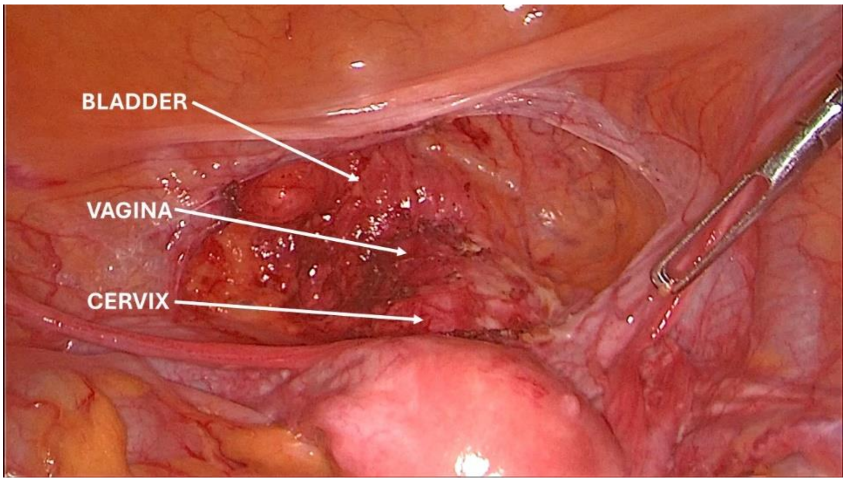

5. Insert the TiLOOP LLS Dubuisson mesh through the trocar.

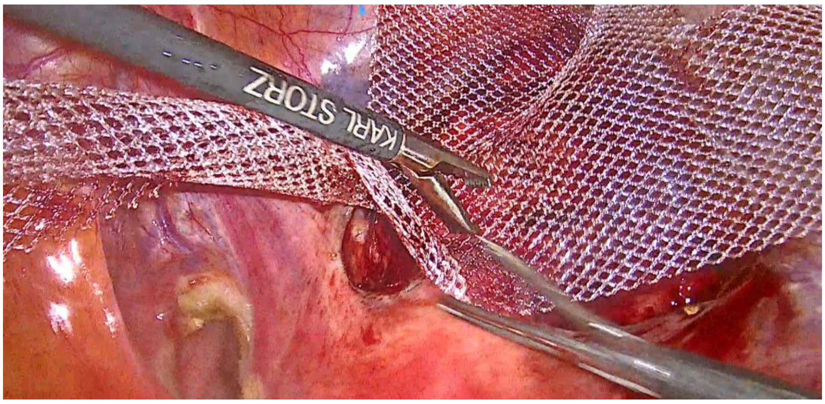

6. Fix the TiLOOP LLS Dubuisson mesh on the anterior aspect of the anterior vaginal wall without folds using resorbable sutures (Vicryl® 2/0 or Polysorb ®2/0). AbsorbaTack™ mechanical fixation sutures (ABSTACK30X) is possible to be used but other stiches are necessary. At cervix is recommended non-absorbable suture (Ethibond® 2/0).

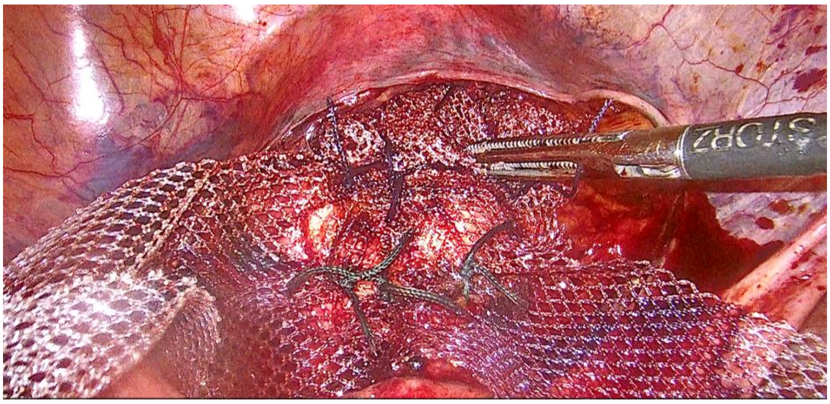

7. Make 3-mm skin incisions 2 cm cranial to the iliac crest and 4 cm posterior to the anterior superior iliac spine on both sides.

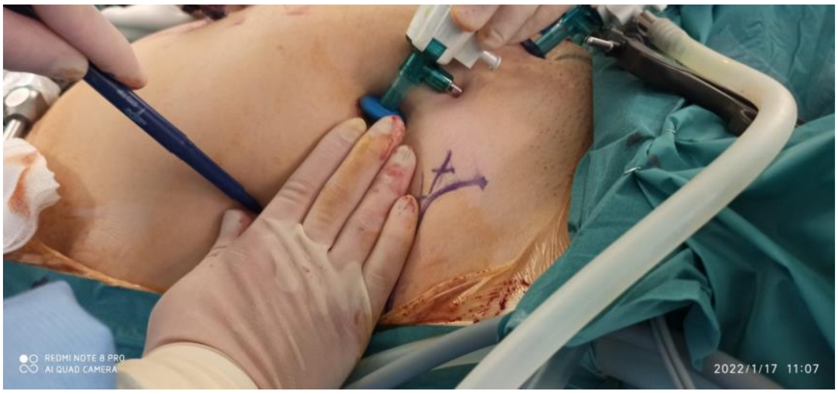

8. Insert forceps vertically through the abdominal wall and aponeurosis to the peritoneum, without perforating it.

9. Advance the clamp pre-peritoneally in the direction of the round ligament and exit below it.

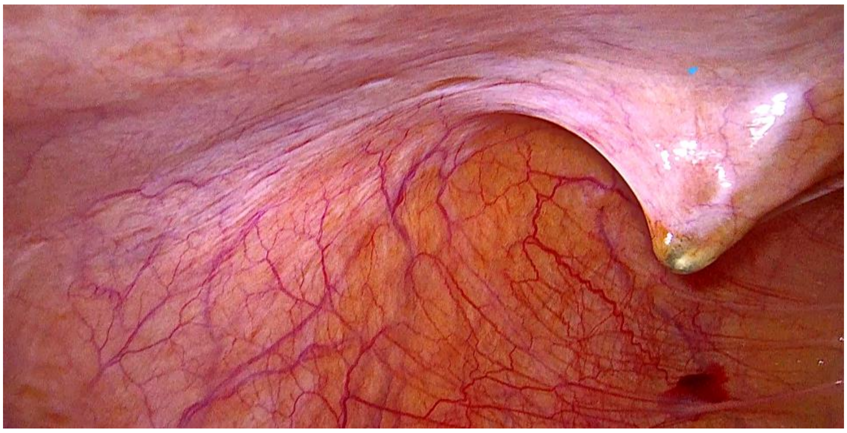

10. Perforate the peritoneum and hold the arm of the mesh. Perform this step bilaterally.

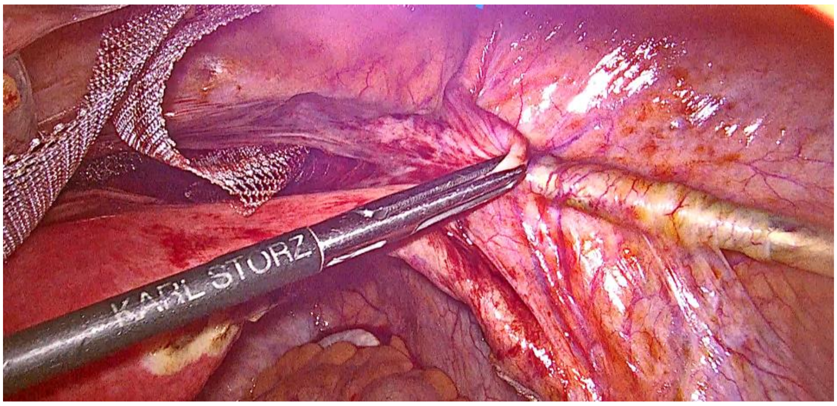

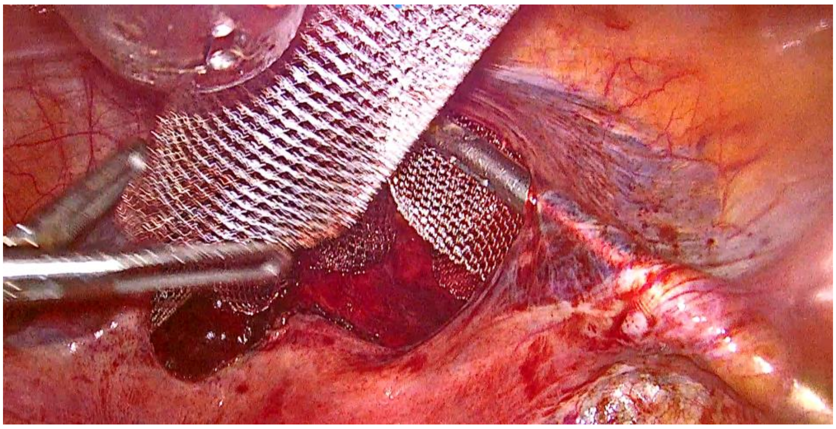

11. Keep both arms of the meshes in the extraperitoneal area, without folds or tension. The uterus returns to its physiological position.

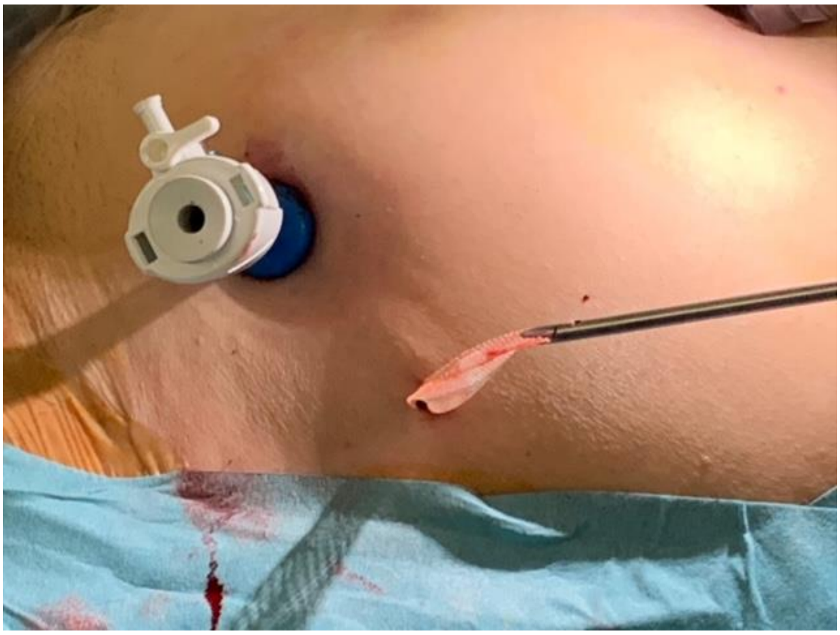

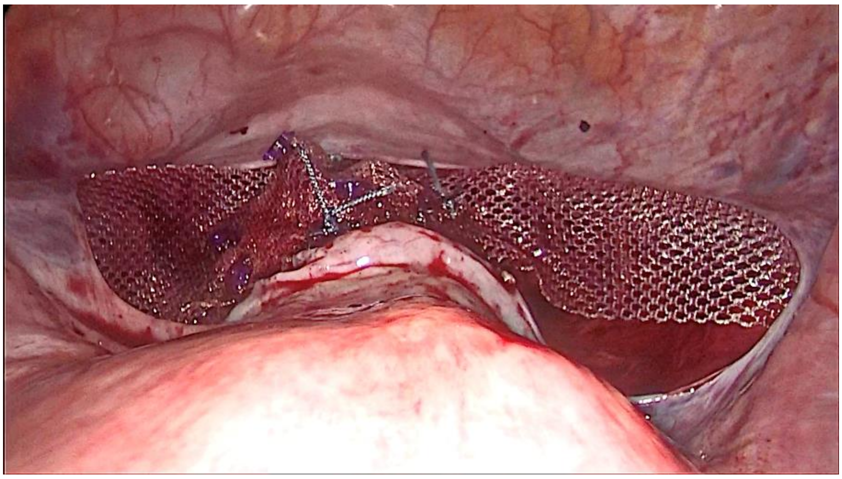

12. Close the peritoneum with resorbable sutures. The end of the mesh is after the pneumoperitoneum is removed. The mesh arms are left unfixed.

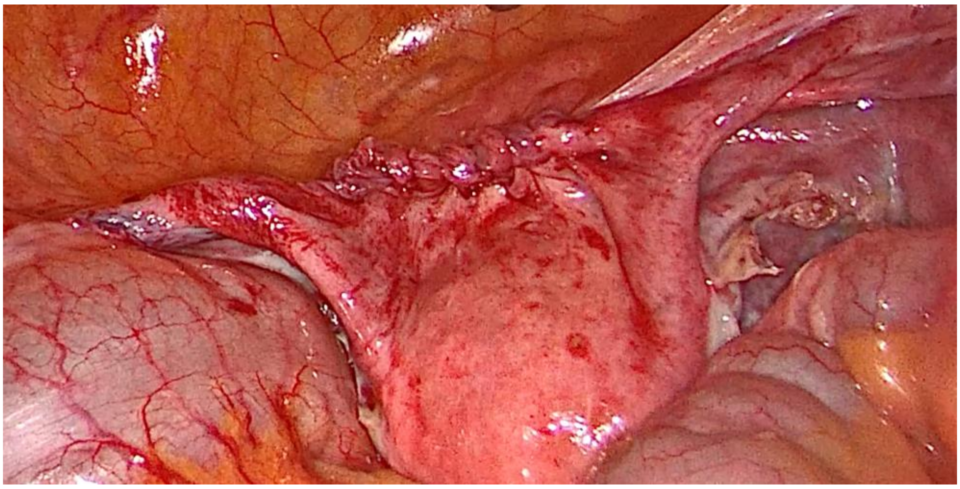

Technique for patients with hysterectomy:

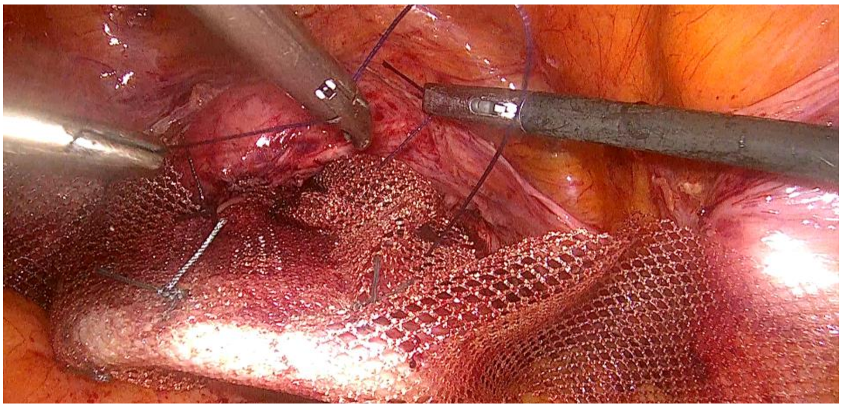

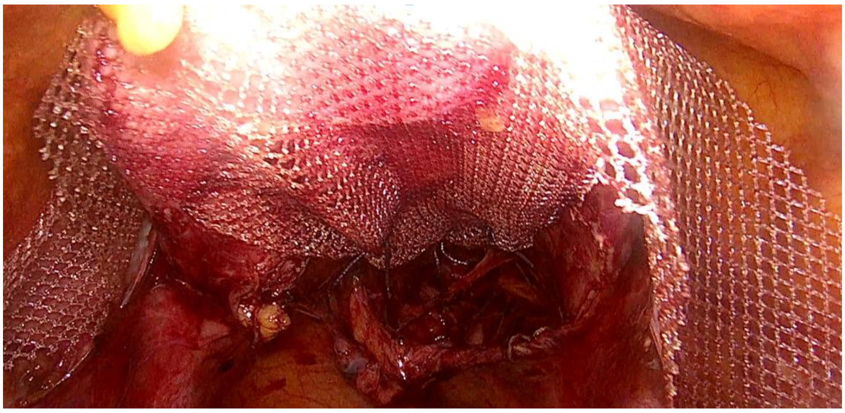

Repair the vesicouterine and rectovaginal space and fix the anterior and posterior portion of the TILOOP LLS H Dubuisson mesh in the anterior vaginal wall, vaginal vault and posterior vaginal wall. The rest of the procedure is the same as that described for hysteropexy.

*******We confirm the authorship of all photographs******

Group B: Cervico/sacrocolpopexy with posterior mesh fixation to the vaginal vault (without deep rectovaginal dissection)

1. PREPARATION OF THE SURGICAL FIELD (same as in Group A)

2. SUPRACERVICAL HYSTERECTOMY

3. DISSECTION OF THE VESICOUTERINE PLICA (same as in Group A)

4. DISSECTION OF THE PROMONTORY

5. PLACEMENT OF THE MESH IN THE VAGINAL DOME OR CERVIX**.** Titanized mesh (TILOOP SCP Y mesh) will be used without bilateral fixation of the levator ani muscle (without posterior mesh fixation on the puborectalis muscle).

As in Group A, it will be important to place the mesh in the anterior part up to the most distal level of vesicovaginal dissection, in the vicinity of the bladder neck, using the resorbable sutures (Vicryl® 2/0 or Polysorb ®2/0) or AbsorbaTack.

At the level of the vaginal vault (or cervix), the mesh will be anchored using 6 AbsorbaTack™ mechanical fixation sutures (ABSTACK30X) or stitches (2 anterior, 2 posterior) and 1 point on each side, with the objective of completely covering the dome or cervix with the mesh. At cervix is recommended non-absorbable suture (Ethibond® 2/0).

6. FIXING THE PROMONTORY It is recommended non-absorbable suture (Ethibond® 2/0) at least 2 stitches.

7. PERITONIZATION Close the peritoneum with resorbable sutures.

When a Hysteropexy is performed the mesh has to be fixed to the posterior cervix and posterior vaginal wall but without fixation in levator ani muscle (without posterior mesh fixation on the puborectalis muscle).
